# GM1 Induced the inflammatory response related to the Raf-1/MEK1/2/ERK1/2 pathway in co-culture of pig mesenchymal stem cells with RAW264.7

**DOI:** 10.1080/19768354.2018.1453546

**Published:** 2018-03-22

**Authors:** Dong Hoon Kwak, You Na Seo, Ju Hyoung Lee, Soon Ju Park, Young Ho Cho, Ji-Su Kim, Sun-Uk Kim, Young-Kug Choo

**Affiliations:** a Institute of Glycosciences, Wonkwang University, Iksan, Republic of Korea; b Institute of Aribio, Sungnam, Republic of Korea; c Department of Biological Science, College of Natural Sciences, Wonkwang University, Iksan, Republic of Korea; d Department of Pharmaceutics and Biotechnology, Medical Engineering College, Konyang University, Daejeon, Republic of Korea; e National Primate Research Center, Korea Institute of Bioscience and Biotechnology, Cheongju, Republic of Korea

**Keywords:** GM1, xenotransplantation, inflammation, MAPK, pig-mesenchymal stem cells

## Abstract

Pig-human xenotransplantation can trigger cell-mediated immune responses. We explored the role of gangliosides in inflammation related to immune rejection in xenotransplantation. Co-culture of xenogeneic cells (pig-MSCs and RAW264.7) was used to emulate xenotransplantation conditions. MTT assay results indicated that cell viability was significantly decreased in pADMSCs co-cultured with RAW264.7 cells. GM1 and GM3 were highly expressed in pADMSCs co-cultured with RAW264.7 cells. pADMSCs co-cultured with RAW264.7 cells strongly expressed pro-inflammatory proteins such as COX-2, iNOS, p50, p65, pIκBα, and TNF-α. GM1-knockdown pADMSCs co-cultured with RAW 264.7 cells did not show significantly altered cell viability, but pro-inflammatory proteins were markedly inhibited. Co-culture of pADMSCs with RAW264.7 cells induced significant phosphorylation (p) of JNK1/2 and pERK1/2. However, pERK1/2 and pJNK1/2 were decreased and MEK1/2 and Raf1 were suppressed in GM1-knockdown pADMSCs co-cultured with RAW264.7 cells. Thus, the Raf-1/MEK1/2/ERK1/2 and JNK1/2 pathways were significantly upregulated in response to increases of GM1 in co-cultured xenogeneic cells. However, the inflammatory response was suppressed in co-culture of GM1-knockdown pADMSCs with RAW264.7 cells via down-regulation of the Raf-1/MEK1/2/ERK1/2 and JNK1/2 pathways. Therefore, the ganglioside GM1 appears to play a major role in the inflammatory response in xenotransplantation via the Raf-1/MEK1/2/ERK1/2 and JNK1/2 pathways.

## Introduction

Gangliosides are complex glycosphingolipids containing one or more sialic acids, and are a main component of cell membranes (Hakomori 1990). Some studies have reported that gangliosides are developmentally controlled in a cell type–specific manner (Yu 1994; Yamamoto et al. 1996; Yu et al. 1988). Additionally, expression of gangliosides is related to the biological processes of stem cells *in vitro* (Kwak et al. 2006).

Mesenchymal stem cells (MSCs) are multipotent cells (Pittenger et al. 1999) that can differentiate into several lineages, including adipocytes, neuron-like cells, osteoblasts, hepatocytes, and myoblasts (Ferrari et al. 1998; Pittenger et al. 1999; Sanchez-Ramos et al. 2000; Hong et al. 2005; Sato et al. 2005; Ryu et al. 2009). Several studies have reported that gangliosides are essential factors in differentiation and proliferation of MSCs (Sanchez-Ramos et al. 2000; Bergante et al. 2014).

Although xenotransplantation has vast clinical potential, it is limited by the problem of immune responses against xenogeneic tissue (Wright et al. 2016). Additionally, xenotransplanted cells, including vascularized organ xenografts, show loss of function within a short time of transplantation in dissonant species combinations. Previous studies reported that gangliosides are related to the inflammatory responses induced in co-culture of xenogeneic cells, such as pig endothelial cells (PAECs) and human leukocytes (Cho et al. 2012). The inflammatory responses were associated with the mitogen-activated protein kinase (MAPK) family (Yin et al. 2016).

The MAPK family of proteins regulates the cell death and the proliferation (Lee et al. 2002; Tarallo and Sordino 2004). The MAPK family consists of two major subgroups: the c-Jun N-terminal stress-activated protein kinase 1/2 (JNK 1/2) subgroup and the extracellular regulated kinase 1/2 (ERK1/2) subgroup (Jung et al. 2004). ERK 1/2, which is activated in inflammatory responses, is related to cell proliferation (Kyriakis and Avruch 2012; Marques-Fernandez et al. 2013).

However, the expression and role of gangliosides in inflammatory responses is unclear, and has not been investigated using xenogeneic co-culture of pig MSCs with cells from other species. In this study, we investigated the role of gangliosides in inflammatory activation using co-culture of pig adipose-derived mesenchymal stem cells (pADMSCs) with RAW 264.7 macrophages.

## Materials and methods

### Culture of pADMSCs and RAW264.7 cells

pADMSCs were provided by the Korea Research Institute of Bioscience and Biotechnology (KRIBB). The cells were cultured in pre-warmed Dulbecco’s Eagle Medium (DMEM) containing 10 ng/ml basic fibroblast growth factor (bGFGF; R&D Systems, Minneapolis, USA), 10% fetal bovine serum (FBS), and 1% (v/v) penicillin/streptomycin (P/S) solution and incubated in a humidified 5% CO_2_ atmosphere at 37°C. RAW 264.7 cells were maintained in DMEM supplemented with 10% FBS and 1% P/S at 37°C in a humidified 5% CO_2_ incubator.

### Co-culture of mini-pig adipose-derived mesenchymal stem cells (pADMSCs) with RAW 264.7 mouse macrophages

pADMSCs were co-cultured with RAW 264.7 cells through seeding of pADMSCs (5 × 10^4^ cells/dish) and RAW 264.7 cells (1 × 10^5^ cells/dish). LPS (from *Escherichia coli* 0111:B4, Sigma) was administered at 10 µM to co-cultured cells and RAW 264.7 cells only.

### Cell viability

Cell proliferation was determined by MTT assay 24 h after initiating culture of pADMSCs and RAW 264.7 cells. pADMSCs co-cultured with RAW 264.7 cells were transferred into 96-well plates at 1 × 10^4^ cells/well and treated with LPS at 10 µM and GM1 synthase siRNA (10 nM), respectively. MTT solution (Sigma) was added to each well and incubated for 4 h and the absorbance was measured at 590 nm using a spectrophotometer.

### Ganglioside extraction and purification

Lee *et al*. have described the methods used to extract and purify gangliosides. Briefly, cells were homogenized in distilled water at 48°C to extract total lipids, which were re-suspended in chloroform/methanol (1:1, v/v), lyophilized using N_2_ gas, and subsequently dissolved in chloroform/methanol/H_2_O (15:30:4, v/v/v). The column was washed with H_2_O to remove non-hydrophobic lipids. Finally, the gangliosides were eluted with methanol, dried at 30°C under N_2_ for 3 h, and stored at −80°C until analysis.

### High-performance thin-layer chromatography

High-performance thin-layer chromatography (HPTLC) analysis of the gangliosides was conducted using a 10 × 10 cm TLC 5651 plate (Merck, Darmstadt, Germany). The purified gangliosides (600 μg protein/lane) were loaded onto TLC 5651 plates that were subsequently developed in chloroform/methanol/0.25% CaCl_2_·H_2_O (50:40:10, v/v/v). The gangliosides were then stained with resorcinol, after which the density of the ganglioside bands was quantified by HPTLC densitometry (ImageJ, NIH). Purified mixed gangliosides (GM3, GM2, GM1, GD3, GD1a, and GD1b) (Matreya LLC, Pleasant Gap, PA, USA) were used as standards.

### Design and selection of allele-specific siRNAs

GM1 and GM3 synthase-specific siRNAs and a control siRNA were synthesized by Bioneer Inc. (Daejeon, Korea). The primers for GM1 were: F, 5′-ATCGCGAGTGTTGCTCTTCGT-3′ and R, 5′-GAGCAACACTGGCACCTGCA-3′. The primers for GM3 were: F, 5′-ATCGGCTAACCTGGACCT-3′ and R, 5′-TACCGTTACCGCAATTCCF-3′. All sequences were confirmed by capillary sequencing. Transfection of siRNAs or DNA vectors was performed using Lipofectamine 3000 reagent (Invitrogen, Carlsbad, USA) according to the manufacturer’s recommendations. The GM1 and GM3 synthase specificity of the siRNAs was determined using HPTLC and western blot analysis to compare the activities of GM1 and GM3.

### Western blot analysis

pADMSCs and RAW 264.7 cells were homogenized in RIPA buffer (Sigma), and then centrifuged at 13,000 rpm for 20 min at 4°C. Proteins (30 µg/lane) were separated on a 10% SDS polyacrylamide gel and then transferred to a nitrocellulose membrane (Hybond ECL; Amersham Pharmacia Biotech, Piscataway, NJ). The blots were blocked for 2 h with 5% bovine serum albumin (BSA) in Tris-buffered saline, and the membrane was incubated for 16 h with the following primary antibodies: BCl-2, Caspase-8, Caspase-9, Caspase-3, and β-actin (1:500; Santa Cruz Biotechnology, Santa Cruz, USA). The blot was then incubated with the corresponding horseradish peroxidase-conjugated secondary antibodies, such as anti-mouse and anti-rabbit (Santa Cruz Biotechnology), and proteins were visualized using the ECL system (Pierce, Rockford, USA).

### Statistical analysis

All data are presented as mean (SD). Multi-group associations were analyzed using one-way ANOVA and two-way ANOVA, followed by Tukey’s and Bonferroni post-hoc pairwise comparisons. A *p-*value < 0.05 was considered statistically significant. All statistical analyses were executed using GraphPad Prism (Ver. 5.00; GraphPad Software Inc., La Jolla, USA).

## Results

### Cell viability and ganglioside expression patterns in pADMSCs, RAW 264.7 cells, and pADMSCs co-cultured with RAW264.7 cells

**Figure 1.**
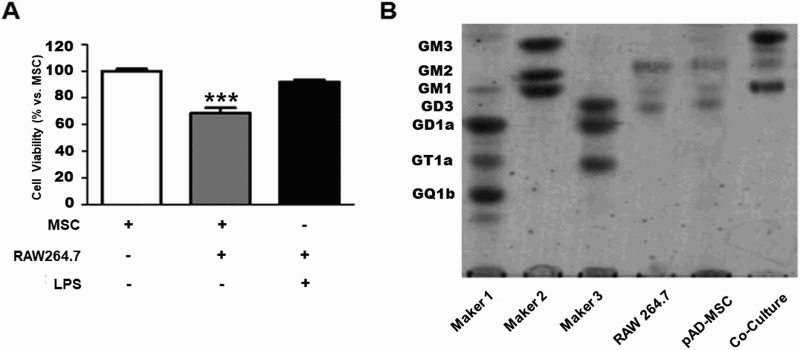
Ganglioside expression patterns and cell viability in GM1-knockdown pADMSCs co-cultured with RAW264.7 cells. (A) Cell viability of pADMSCs co-cultured with RAW264.7 cells. (B) Expression of gangliosides as detected by HPTLC in co-culture of GM1-knockdown pADMSCs with RAW264.7. ***p < 0.001 indicates a significant difference from the pADMSCs.

The co-culture was designed to emulate the conditions of xenograft. The cell culture groups consisted of pADMSCs only, RAW264.7 cells plus LPS, and co-culture of pADMSCs with RAW264.7 cells. Figure 1 shows cell viability as determined by MTT assays. Cell viability was significantly decreased when pADMSCs were co-cultured with RAW264.7 cells (Figure 1(A)). However, cell viability was similar to control in LPS only group (Figure 1(A)). In addition, we examined the ganglioside expression profile in pADMSCs only, RAW264.7 cells only, and pADMSCs co-cultured with RAW264.7 cells. GM2 and GD3 were weakly expressed in pADMSCs only and RAW264.7 cells only (Figure 1(B)). However, GM1 and GM3 were highly expressed in co-culture of pADMSCs with RAW264.7 cells (Figure 1(B)).

### Expression of pro-inflammatory factors in pADMSCs co-cultured with RAW264.7 cells

**Figure 2.**
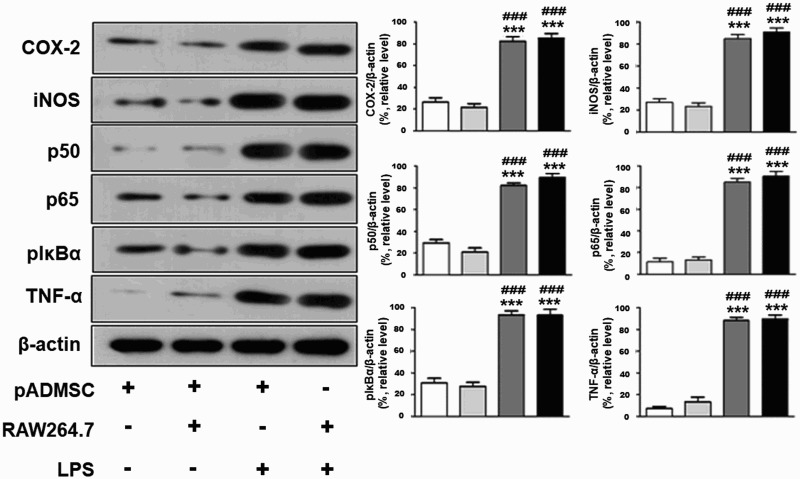
Increase of pro-inflammatory factors in co-culture of xenogeneic cells (pADMSCs with RAW264.7). Expression of β-actin and pro-inflammatory factors such as COX-2, iNOS, p50, p65, pIκbα, and TNF-α was detected by western blotting. ***p < 0.001 indicates a significant difference from pADMSCs. **^###^***p* < 0.001 indicates a significant difference from RAW264.7 cells.

We investigated expression levels of pro-inflammatory factors including COX-2, iNOS, p50, p65, pIκBα, and TNF-α. Pro-inflammatory factors were very weakly expressed in pADMSCs and RAW264.7 cells (Figure 2). Conversely, RAW264.7 cells plus LPS, the positive control, showed significantly increased expression of pro-inflammatory factors (Figure 2, *p *< 0.001). Pro-inflammatory proteins, such as COX-2, iNOS, p50, p65, pIκBα, and TNF-α, were strongly expressed in pADMSCs co-cultured with RAW264.7 cells, compared with pADMSCs only and RAW264.7 cells only (Figure 2, *p *< 0.001).

### Cell viability in pADMSCs co-cultured with RAW 264.7 cells with knockdown of GM1 and GM3 synthase using siRNA

**Figure 3.**
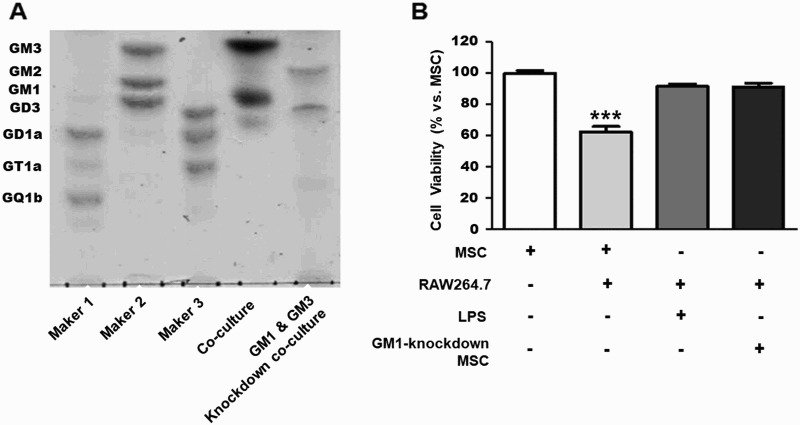
Expression of the ganglioside GM1 and cell viability in GM1-knockdown pADMSCs co-cultured with RAW264.7 cells. (A) Knockdown of GM1 and GM3 was detected by HPTLC in co-culture of GM1-knockdown pADMSCs with RAW264.7 cells. (B) Cell viability of GM1-knockdown pADMSCs co-cultured with RAW264.7 cells. ***p < 0.001 indicates a significant difference from pADMSCs co-cultured with RAW264.7 cells.

We investigated the effects of GM1 and GM3 knockdown in pADMSCs using GM1 and GM3 synthase siRNA. Figure 3(A) shows the results of GM1 and GM3 knockdown in co-culture of pADMSCs with RAW264.7 cells (Figure 3(A)). Cell viability significantly decreased in pADMSC co-cultured with RAW264.7 cells as a positive control (Figure 3(B)). However, cell viability was significantly higher in pADMSCs (GM1 synthase knockdown) co-cultured with RAW264.7 cells than in pADMSCs co-cultured with RAW264.7 cells (Figure 3(B)).

### Inhibition of expression of pro-inflammatory factors by knockdown of GM1 and GM3 synthase using siRNA

**Figure 4.**
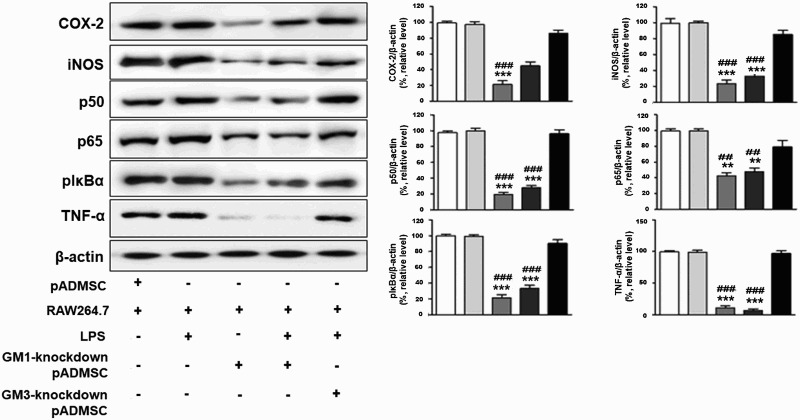
Inhibition of pro-inflammatory factors in co-culture of GM1-knockdown pADMSCs with RAW264.7 cells. Expression of β-actin and pro-inflammatory factors such as COX-2, iNOS, p50, p65, pIκbα, and TNF-α was detected by western blotting. ***p < 0.001 indicates a significant difference from co-culture of pADMSCs with RAW264.7. **^###^***p* < 0.001 indicates a significant difference from RAW264.7 cells treated with LPS.

We examined inhibition of expression of pro-inflammatory factors, including COX-2, iNOS, p50, p65, pIκBα, and TNF-α. The pro-inflammatory factors were significantly expressed in pADMSCs co-cultured with RAW264.7 cells and RAW264.7 cells plus LPS (Figure 4). In contrast, pro-inflammatory factors such as COX-2, iNOS, p50, p65, pIκBα, and TNF-α were markedly inhibited in GM1-knockdown pADMSCs co-cultured with RAW 264.7 cells (Figure 4). Moreover, pro-inflammatory factors were meanly inhibited in GM1-knockdown pADMSCs co-cultured with RAW264.7 cells plus LPS (Figure 4). However, expression of pro-inflammatory factors was significantly increased in GM3-knockdown pADMSCs.co-cultured with RAW264.7 cells plus LPS.

### Involvement of the MAPK pathway with GM1 in inflammation of co-cultured pADMSCs and RAW264.7 cells

**Figure 5.**
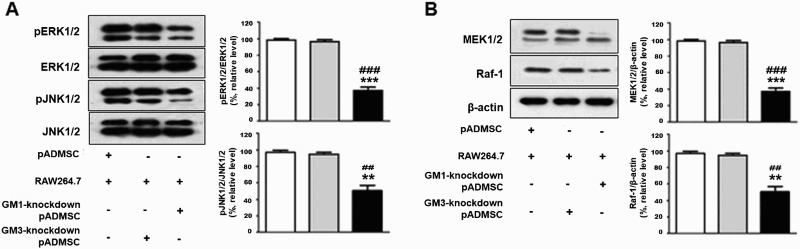
Suppression of Raf-1/MEK1/2ERK1/2 phosphorylation and JNK1/2 in co-culture of GM1-knockdown pADMSCs with RAW264.7. (**A**) β-actin, total ERK1/2, phosphorylated ERK1/2 (pERK1/2), total JNK1/2, and pJNK1/2, and (**B**) MEK1/2 and Raf-1 were detected by western blotting. ***p < 0.001 vs co-culture of pADMSCs with RAW264.7. **^###^***p* < 0.001 vs RAW264.7 cells treated with LPS.

We attempted to determine the role of MAPK and to elucidate its mechanism of action in co-culture of pADMSCs with RAW264.7 cells with or without knockdown of GM1. As shown in Figure 5(A), co-culture of pADMSCs with RAW264.7 cells induced significant phosphorylation (p) of pJNK1/2 and pERK1/2. However, pERK1/2 and pJNK1/2 were markedly decreased in GM1-knockdown pADMSCs co-cultured with RAW264.7 cells compared with co-culture of pADMSCs and RAW264.7 cells (Figure 5(A)). In addition, we investigated the upstream ERK1/2 signaling, including mitogen-activated protein kinase 1/2 (MEK1/2) and Raf1. MEK1/2 and Raf1 were significantly activated in co-culture of pADMSCs and RAW264.7 cells (Figure 5(B)). Moreover, activation of MEK1/2 and Raf1 was strongly increased in GM3-knockdown pADMSCs. However, in co-culture of GM1-knockdown pADMSCs with RAW264.7 cells, MEK1/2 and Raf1 were meanly decreased compared with co-culture of pADMSCs with RAW264.7 cells plus LPS (Figure 5(B)). These results indicated that GM1 inhibited both pathways (Raf1/MEK1/ERK1/2 pathway and JNK1/2 pathway) in inflammation induced by co-culture of pADMSCs and RAW264.7 cells.

## Discussion

Inflammation results from an excessive immune response, as part of the mechanism for protection against damaged tissue and foreign substances (Fitzpatrick 2001). Inflammatory mediators such as NO are produced by macrophages (Lee et al. 2016). Inducible iNOS and COX-2 are expressed following increased production of NO in LPS-stimulated macrophages (Zhao et al. 2013). iNOS is an important factor for the development of inflammation and subsequent maintenance of the inflammatory response. COX-2 is another important factor in inflammation (Lee et al. 2009). In addition, the activities of iNOS and COX-2 related to production of TNF-α play important roles in inflammatory responses (Shin et al. 2015). NF-κB is an important transcription factor associated with inflammatory responses, which induces the expression of various inflammatory factors, including iNOS, COX-2, and TNF-α (An et al. 2011; Yamada et al. 2014). In LPS-stimulated RAW264.7 macrophage cells, NF-κB is activated by the protein I-κBα (Ramaswami et al. 2012). Some reports have shown that gangliosides can induce production of cyclooxygenase-2. In this study, we observed that pro-inflammatory factors including COX-2, iNOS, p65, p50, p-IκBα, and TNF-α were significantly increased in pADMSCs co-cultured with RAW264.7 cells (Figure 2). Moreover, the increases in pro-inflammatory factors in co-culture of pADMSCs with RAW 264.7 cells were similar to the pro-inflammatory factor expression observed in LPS-stimulated RAW 264.7 cells, as a positive control (Figure 2). However, pro-inflammatory factors were significantly suppressed in GM1-knockdown pADMSCs co-cultured with macrophages (Figure 2). These results indicated that in co-culture of xenogeneic cells (pig MSCs with RAW264.7 mouse macrophages) inflammation related to the rejection of xenografts was mediated by GM1.

Several studies have reported that phosphorylation of three MAPKs (ERK, JNK, and p38) occurs by NF-κB activation (Hwang et al. 2011; Li et al. 2011). Szelenyi and Uros reported that the ERK1/2 pathway is a dominant and highly responsive pathway in inflammation (Szelenyi and Urso 2012). MEK-mediated ERK activation is the most important regulatory step in inflammation (Parthasarathy and Philipp 2014). A previous study indicated that the anti-inflammatory mechanism of flavonoids was related to inhibition of ERK phosphorylation by down-regulation of the expression of iNOS and COX-2 (Han et al. 2013), but was independent of the JNK and P38 pathway (Mazier et al. 2001). In addition, another report indicated that GM1 can activate ERKs in young rats and that GM1 induces activation of ERK1/2 by the Raf-1/MEK1/2 pathway in the VSMCs pathway (Duchemin et al. 2002). Ceramide, an important component of gangliosides, is known to be related to the ERK1/2 and the JNK pathways (Maziere et al. 2001). In particular, ceramide regulates the ERK1/2 pathway via activated RAf-1 and MEKs in various cell types (Willaime et al. 2001). In this study, we investigated how the MAPK pathway is involved with inflammation in pADMSCs co-cultured with RAW264.7 macrophage cells, and we found that expression of ERK1/2 and JNK1/2 were meanly activated in pADMSCs co-cultured with RAW264.7 cells (Figure 5(A)). Furthermore, activation of MEK1/2 and Raf-1, the up-stream pathway of ERK1/2, was strongly increased in pADMSCs co-cultured with RAW 264.7 cells (Figure 5(B)). However, other components of the MAPK pathway, including ERK1/2, JNK1/2, MEK1/2, and Raf-1, were significantly suppressed in co-culture of pADMSCs with GM1 knockdown (Figure 5). These results indicated that ERK1/2 phosphorylation by upregulation of MEK1/2/Raf-1 was associated with macrophage inflammation mediated by an increase of the ganglioside GM1 in co-culture of xenogeneic cells (pig MSCs with RAW264.7 mouse macrophages).

## Conclusion

Cell - mediated immune responses can induce by xenotransplantation of pig and human. We explored the role of gangliosides in inflammation related to immune rejection in xenotransplantation. We used the co-culture of xenogeneic cells (pig-MSCs and RAW264.7) for emulate xenotransplantation conditions. We observed the highly expression of GM1 and GM3 when pADMSCs co-cultured with RAW264.7 cells. Pro-inflammatory proteins such as COX-2, iNOS, p50, p65, pIκBα, and TNF-α strongly expressed when pADMSCs co-cultured with RAW264.7 cells. In GM1-knockdown pADMSCs co-cultured with RAW 264.7 cells, pro-inflammatory proteins were markedly inhibited. In addition, we observed the phosphorylation (p) of JNK1/2 and pERK1/2 was significant induced in co-culture of pADMSCs with RAW264.7 cells. However, pERK1/2, pJNK1/2, MEK1/2 and Raf1 were suppressed in GM1-knockdown pADMSCs co-cultured with RAW264.7 cells. Thus, GM1 increases significantly up regulated the Raf-1/MEK1/2/ERK1/2 and JNK1/2 pathways in co-cultured xenogeneic cells. However, we find that inflammatory response suppressed by down-regulation of the Raf-1/MEK1/2/ERK1/2 and JNK1/2 pathways in co-culture of GM1-knockdown pADMSCs with RAW264.7 cells. Therefore, the ganglioside GM1 appears to play a major role in the inflammatory response in xenotransplantation via the Raf-1/MEK1/2/ERK1/2 and JNK1/2 pathways.

## References

[CIT0001] An HJ, Kim IT, Park HJ, Kim HM, Choi JH, Lee KT. 2011 Tormentic acid, a triterpenoid saponin, isolated from Rosa rugosa, inhibited LPS-induced iNOS, COX-2, and TNF-alpha expression through inactivation of the nuclear factor-kappab pathway in RAW 264.7 macrophages. International Immunopharmacology. 11:504–510. doi: 10.1016/j.intimp.2011.01.002 21237302

[CIT0002] Bergante S, Torretta E, Creo P, Sessarego N, Papini N, Piccoli M, Fania C, Cirillo F, Conforti E, Ghiroldi A, et al. 2014 Gangliosides as a potential new class of stem cell markers: the case of GD1a in human bone marrow mesenchymal stem cells. Journal of Lipid Research. 55:549–560. doi: 10.1194/jlr.M046672 24449473 PMC3934739

[CIT0003] Cho JH, Kim JS, Lim MU, Min HK, Kwak DH, Ryu JS, Lee JT, Kim SU, Kim CH, Kim CH, et al. 2012 Human leukocytes regulate ganglioside expression in cultured micro-pig aortic endothelial cells. Laboratory Animal Research. 28:255–263. doi: 10.5625/lar.2012.28.4.255 23326286 PMC3542384

[CIT0004] Duchemin AM, Ren Q, Mo L, Neff NH, Hadjiconstantinou M. 2002 GM1 ganglioside induces phosphorylation and activation of Trk and Erk in brain. Journal of Neurochemistry. 81:696–707. doi: 10.1046/j.1471-4159.2002.00831.x 12065629

[CIT0005] Ferrari G, Cusella-De Angelis G, Coletta M, Paolucci E, Stornaiuolo A, Cossu G, Mavilio F. 1998 Muscle regeneration by bone marrow-derived myogenic progenitors. Science. 279:1528–1530. Epub 1998/03/21. doi: 10.1126/science.279.5356.1528 9488650

[CIT0006] Fitzpatrick FA. 2001 Inflammation, carcinogenesis and cancer. International Immunopharmacology. 1:1651–1667. doi: 10.1016/S1567-5769(01)00102-3 11562058

[CIT0007] Hakomori S. 1990 Bifunctional role of glycosphingolipids. Modulators for transmembrane signaling and mediators for cellular interactions. Journal of Biological Chemistry. 265:18713–18716. Epub 1990/11/05. 2229037

[CIT0008] Han S, Lee JH, Kim C, Nam D, Chung WS, Lee SG, Ahn KS, Cho SK, Cho M, Ahn KS. 2013 Capillarisin inhibits iNOS, COX-2 expression, and proinflammatory cytokines in LPS-induced RAW 264.7 macrophages via the suppression of ERK, JNK, and NF-kappaB activation. Immunopharmacology and Immunotoxicology. 35:34–42. doi: 10.3109/08923973.2012.736522 23131135

[CIT0009] Hong SH, Gang EJ, Jeong JA, Ahn C, Hwang SH, Yang IH, Park HK, Han H, Kim H. 2005 In vitro differentiation of human umbilical cord blood-derived mesenchymal stem cells into hepatocyte-like cells. Biochemical and Biophysical Research Communications. 330:1153–1161. Epub 2005/04/13. doi: 10.1016/j.bbrc.2005.03.086 15823564

[CIT0010] Hwang MH, Damte D, Lee JS, Gebru E, Chang ZQ, Cheng H, Jung BY, Rhee MH, Park SC. 2011 Mycoplasma hyopneumoniae induces pro-inflammatory cytokine and nitric oxide production through NFkappaB and MAPK pathways in RAW264.7 cells. Veterinary Research Communications. 35:21–34. doi: 10.1007/s11259-010-9447-5 21104123

[CIT0011] Jung YS, Lee DH, Lim H, Yi KY, Yoo SE, Kim E. 2004 KR-31378 protects cardiac H9c2 cells from chemical hypoxia-induced cell death via inhibition of JNK/p38 MAPK activation. The Japanese Journal of Physiology. 54:575–583. doi: 10.2170/jjphysiol.54.575 15760490

[CIT0012] Kwak DH, Yu K, Kim SM, Lee DH, Jung JU, Seo JW, Kim N, Lee S, Jung KY, You HK, et al. 2006 Dynamic changes of gangliosides expression during the differentiation of embryonic and mesenchymal stem cells into neural cells. Experimental & Molecular Medicine. 38:668–676. Epub 2007/01/05. doi: 10.1038/emm.2006.79 17202843

[CIT0013] Kyriakis JM, Avruch J. 2012 Mammalian MAPK signal transduction pathways activated by stress and inflammation: a 10-year update. Physiological Reviews. 92:689–737. doi: 10.1152/physrev.00028.2011 22535895

[CIT0014] Lee DH, Kim NR, Lim BS, Lee YK, Yang HC. 2009 Effects of TEGDMA and HEMA on the expression of COX-2 and iNOS in cultured murine macrophage cells. Dental Materials: Official Publication of the Academy of Dental Materials. 25:240–246. doi: 10.1016/j.dental.2008.05.014 18755506

[CIT0016] Lee S, Lee HS, Baek M, Lee DY, Bang YJ, Cho HN, Lee YS, Ha JH, Kim HY, Jeoung DI. 2002 MAPK signaling is involved in camptothecin-induced cell death. Molecules and Cells. 14:348–354. 12521296

[CIT0017] Lee SH, Kwak CH, Lee SK, Ha SH, Park J, Chung TW, Ha KT, Suh SJ, Chang YC, Chang HW, et al. 2016 Anti-Inflammatory effect of ascochlorin in LPS-stimulated RAW 264.7 macrophage cells Is accompanied With the down-regulation of iNOS, COX-2 and proinflammatory cytokines through NF-kappaB, ERK1/2, and p38 signaling pathway. Journal of Cellular Biochemistry. 117:978–987. doi: 10.1002/jcb.25383 26399466

[CIT0018] Li M, Wu ZM, Yang H, Huang SJ. 2011 NFkappab and JNK/MAPK activation mediates the production of major macrophage- or dendritic cell-recruiting chemokine in human first trimester decidual cells in response to proinflammatory stimuli. The Journal of Clinical Endocrinology and Metabolism. 96:2502–2511. doi: 10.1210/jc.2011-0055 21677045 PMC3146787

[CIT0019] Marques-Fernandez F, Planells-Ferrer L, Gozzelino R, Galenkamp KM, Reix S, Llecha-Cano N, Lopez-Soriano J, Yuste VJ, Moubarak RS, Comella JX. 2013 TNFalpha induces survival through the FLIP-L-dependent activation of the MAPK/ERK pathway. Cell Death & Disease. 4:e493. doi: 10.1038/cddis.2013.25 23412386 PMC3734812

[CIT0020] Maziere C, Conte MA, Leborgne L, Levade T, Hornebeck W, Santus R, Maziere JC. 2001 UVA radiation stimulates ceramide production: relationship to oxidative stress and potential role in ERK, JNK, and p38 activation. Biochemical and Biophysical Research Communications. 281:289–294. doi: 10.1006/bbrc.2001.4348 11181043

[CIT0021] Parthasarathy G, Philipp MT. 2014 The MEK/ERK pathway is the primary conduit for Borrelia burgdorferi-induced inflammation and P53-mediated apoptosis in oligodendrocytes. Apoptosis: An International Journal on Programmed Cell Death. 19:76–89. doi: 10.1007/s10495-013-0913-8 24114360 PMC3947362

[CIT0022] Pittenger MF, Mackay AM, Beck SC, Jaiswal RK, Douglas R, Mosca JD, Moorman MA, Simonetti DW, Craig S, Marshak DR. 1999 Multilineage potential of adult human mesenchymal stem cells. Science. 284:143–147. Epub 1999/04/02. doi: 10.1126/science.284.5411.143 10102814

[CIT0023] Ramaswami S, Manna S, Juvekar A, Kennedy S, Vancura A, Vancurova I. 2012 Chromatin immunoprecipitation analysis of NFkappaB transcriptional regulation by nuclear IkappaBalpha in human macrophages. Methods in Molecular Biology. 809:121–134. doi: 10.1007/978-1-61779-376-9_8 22113272

[CIT0024] Ryu JS, Ko K, Lee JW, Park SB, Byun SJ, Jeong EJ, Choo YK. 2009 Gangliosides are involved in neural differentiation of human dental pulp-derived stem cells. Biochemical and Biophysical Research Communications. 387:266–271. Epub 2009/07/08. doi: 10.1016/j.bbrc.2009.07.005 19580786

[CIT0025] Sanchez-Ramos J, Song S, Cardozo-Pelaez F, Hazzi C, Stedeford T, Willing A, Freeman TB, Saporta S, Janssen W, Patel N, et al. 2000 Adult bone marrow stromal cells differentiate into neural cells in vitro. Experimental Neurology. 164:247–256. Epub 2000/08/01. doi: 10.1006/exnr.2000.7389 10915564

[CIT0026] Sato Y, Araki H, Kato J, Nakamura K, Kawano Y, Kobune M, Sato T, Miyanishi K, Takayama T, Takahashi M, et al. 2005 Human mesenchymal stem cells xenografted directly to rat liver are differentiated into human hepatocytes without fusion. Blood. 106:756–763. Epub 2005/04/09. doi: 10.1182/blood-2005-02-0572 15817682

[CIT0027] Shin JS, Hong Y, Lee HH, Ryu B, Cho YW, Kim NJ, Jang DS, Lee KT. 2015 Fulgidic acid isolated from the rhizomes of cyperus rotundus suppresses LPS-induced iNOS, COX-2, TNF-alpha, and IL-6 expression by AP-1 inactivation in RAW264.7 macrophages. Biological & Pharmaceutical Bulletin. 38:1081–1086. doi: 10.1248/bpb.b15-00186 26133719

[CIT0028] Szelenyi ER, Urso ML. 2012 Time-course analysis of injured skeletal muscle suggests a critical involvement of ERK1/2 signaling in the acute inflammatory response. Muscle & Nerve. 45:552–561. doi: 10.1002/mus.22323 22431089

[CIT0029] Tarallo R, Sordino P. 2004 Time course of programmed cell death in ciona intestinalis in relation to mitotic activity and MAPK signaling. Developmental Dynamics: An Official Publication of the American Association of Anatomists. 230:251–262. doi: 10.1002/dvdy.20055 15162504

[CIT0030] Willaime S, Vanhoutte P, Caboche J, Lemaigre-Dubreuil Y, Mariani J, Brugg B. 2001 Ceramide-induced apoptosis in cortical neurons is mediated by an increase in p38 phosphorylation and not by the decrease in ERK phosphorylation. The European Journal of Neuroscience. 13:2037–2046. doi: 10.1046/j.0953-816x.2001.01581.x 11422444

[CIT0031] Wright K, Dziuk R, Mital P, Kaur G, Dufour JM. 2016 Xenotransplanted Pig sertoli cells inhibit both the alternative and classical pathways of complement-mediated cell lysis while pig islets are killed. Cell Transplantation. 25:2027–2040. doi: 10.3727/096368916X692032 27305664 PMC6126928

[CIT0032] Yamada H, Kikuchi S, Inui T, Takahashi H, Kimura K, Scavone C. 2014 Gentiolactone, a secoiridoid dilactone from Gentiana triflora, inhibits TNF-alpha, iNOS and Cox-2 mRNA expression and blocks NF-kappaB promoter activity in murine macrophages. PloS One. 9:e113834. doi: 10.1371/journal.pone.0113834 25423092 PMC4244148

[CIT0033] Yamamoto A, Haraguchi M, Yamashiro S, Fukumoto S, Furukawa K, Takamiya K, Atsuta M, Shiku H. 1996 Heterogeneity in the expression pattern of two ganglioside synthase genes during mouse brain development. Journal of Neurochemistry. 66:26–34. Epub 1996/01/01. doi: 10.1046/j.1471-4159.1996.66010026.x 8522963

[CIT0034] Yin N, Qi X, Tsai S, Lu Y, Basir Z, Oshima K, Thomas JP, Myers CR, Stoner G, Chen G. 2016 P38gamma MAPK is required for inflammation-associated colon tumorigenesis. Oncogene. 35:1039–1048. doi: 10.1038/onc.2015.158 25961922

[CIT0035] Yu RK. 1994 Development regulation of ganglioside metabolism. Progress in Brain Research. 101:31–44. Epub 1994/01/01. doi: 10.1016/S0079-6123(08)61938-X 8029460

[CIT0036] Yu RK, Macala LJ, Taki T, Weinfield HM, Yu FS. 1988 Developmental changes in ganglioside composition and synthesis in embryonic rat brain. Journal of Neurochemistry. 50:1825–1829. Epub 1988/06/01. doi: 10.1111/j.1471-4159.1988.tb02484.x 3131485

[CIT0037] Zhao F, Chen L, Bi C, Zhang M, Jiao W, Yao X. 2013 In vitro anti-inflammatory effect of picrasmalignan A by the inhibition of iNOS and COX2 expression in LPSactivated macrophage RAW 264.7 cells. Molecular Medicine Reports. 8:1575–1579. doi: 10.3892/mmr.2013.1663 24002245

